# Community metabolism in a deep (stratified) tropical reservoir during a period of high water-level fluctuations

**DOI:** 10.1007/s10661-014-3870-y

**Published:** 2014-07-05

**Authors:** Patricia M. Valdespino-Castillo, Martín Merino-Ibarra, Jorge Jiménez-Contreras, Fermín S. Castillo-Sandoval, Jorge A. Ramírez-Zierold

**Affiliations:** 1 Posgrado en Ciencias del Mar y Limnología, Universidad Nacional Autónoma de México, Circuito Exterior s/n, Ciudad Universitaria, México, 04510 México; 2 Unidad Académica de Ecología y Biodiversidad Acuática, Instituto de Ciencias del Mar y Limnología, Universidad Nacional Autónoma de México, Circuito Exterior s/n, Ciudad Universitaria, México, 04510 México

**Keywords:** Boundary mixing, Eutrophic, Heterotrophy, Nutrients, Respiration, Silicate

## Abstract

As long as lakes and reservoirs are an important component of the global carbon cycle, monitoring of their metabolism is required, especially in the tropics. In particular, the response of deep reservoirs to water-level fluctuations (WLF) is an understudied field. Here, we study community metabolism through oxygen dynamics in a deep monomictic reservoir where high WLF (~10 m) have recently occurred. Simultaneous monitoring of environmental variables and zooplankton dynamics was used to assess the effects of WLF on the metabolism of the eutrophic Valle de Bravo (VB) reservoir, where cyanobacteria blooms are frequent. Mean gross primary production (*P*_g_) was high (2.2 g C m^−2^ day^−1^), but temporal variation of *P*_g_ was low except for a drastic reduction during circulation attributed to zooplankton grazing. The trophogenic layer showed net autotrophy on an annual basis, but turned to net heterotrophy during mixing, and furthermore when the whole water-column oxygen balance was calculated, considering the aphotic respiration (*R*_aphotic_). The high total respiration resulting (3.1 g C m^−2^ day^−1^) is considered to be partly due to mixing enhanced by WLF. Net ecosystem production was equivalent to a net export of 3.4 mg CO_2_ m^−2^ day^−1^ to the atmosphere. Low water levels are posed to intensify boundary-mixing events driven by the wind during the stratification in VB. Long-term monitoring showed changes in the planktonic community and a strong silicon decrease that matched with low water-level periods. The effects of low water-level on metabolism and planktonic community in VB suggest that water-level manipulation could be a useful management tool to promote phytoplankton groups other than cyanobacteria.

## Introduction

Recent assessments show that freshwater ecosystems constitute a significant component of the global carbon cycle that deserves attention (Cole et al. 2007). In contrast, studies on carbon fluxes and community metabolism are scarce, particularly in tropical lakes and reservoirs (St. Louis et al. 2000; Staehr et al. 2012). The urgency to monitor these processes in lakes and reservoirs is further stressed because their contribution has changed significantly as a result of human activities and it is expected that it will continue changing in the future in response to climate change coupled with an increase in the number of impoundments (St. Louis et al. 2000; Tranvik et al. 2009). In many of these systems, eutrophication is an emergent problem (Downing et al. 1999) due to the increased nutrient inputs. An imbalance between primary production and organic matter catabolism is often favored, modifying C fluxes and causing extended anoxic zones (Mee 2006). The severity of eutrophication depends on the capacity of each system to replenish the oxygen depleted by respiration. When the combined action of both biological and physical processes cannot replenish the oxygen consumed within an aquatic system, a level of “critical eutrophication” is reached (Mee 1988) and most biogeochemical cycles are drastically altered. On account of their relatively reduced surface, deep lakes and reservoirs are therefore more vulnerable than shallow ones.

Many lakes and reservoirs are also increasingly affected by water-level fluctuations (WLF, Wantzen et al. 2008). WLF affect the ecological processes and patterns of water systems in multiple ways (Naselli-Flores and Barone 1997; Wantzen et al. 2008; Mac Donagh et al. 2009). Because depth is highly influent on the mixing regime of water bodies, extreme reduction of water levels may convert monomictic lakes to polymictic ones (Zohary and Ostrovsky 2011). Through their effect on mixing processes, WLF can also cause rapid modifications of biogeochemical processes, oxygen dynamics, and the metabolic balance. Important variations in plankton communities have also been associated to WLF (Naselli-Flores and Barone 1997; Mac Donagh et al. 2009; Wang et al. 2011). Due to this, WLF have also a potential application for reservoir management that needs to be explored (Geraldes and Boavida 2005). Studies on the effect of WLF are scarce and restricted to Europe and Australia (Mac Donagh et al. 2009). When reviewing current knowledge on the effects of WLF, Zohary and Ostrovsky (2011) concluded that “the response of aquatic ecosystems, particularly deep lakes, to water-level fluctuations is an under-studied field of crucial importance to the management of water resources”.

In this paper, we monitor community oxygen metabolism in the tropical, deep and eutrophic reservoir of Valle de Bravo (VB), where high WLF (~10 m) have occurred recently due to its intensive use as water source for Mexico City. Our aim is to contribute to the understanding of biogeochemical processes in freshwater ecosystems, to assess how WLF can affect planktonic production and respiration in deep eutrophic water bodies, and to outline the importance of long term monitoring of metabolism in water bodies affected by human activities.

## Study area

**Fig. 1:**
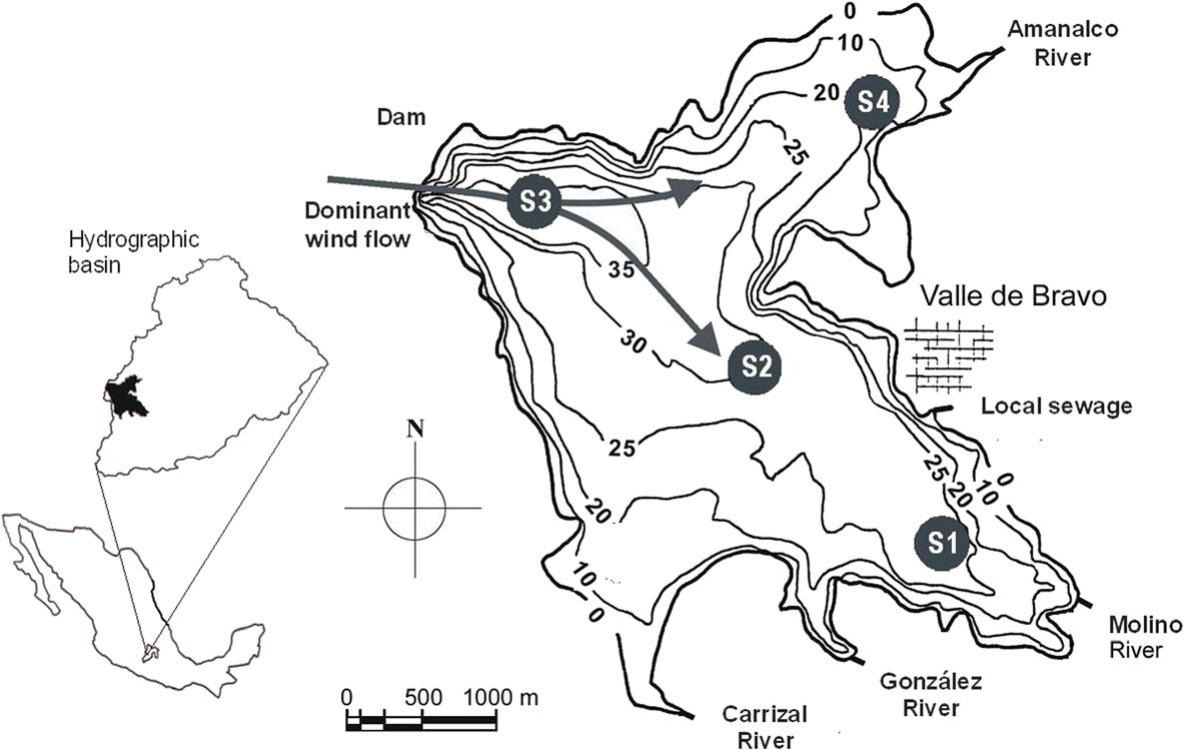
Location and bathymetry of the VB reservoir. The *circles* show the location of the sampling stations. Depth contours in meters below the capacity level of the reservoir

**Fig. 2:**
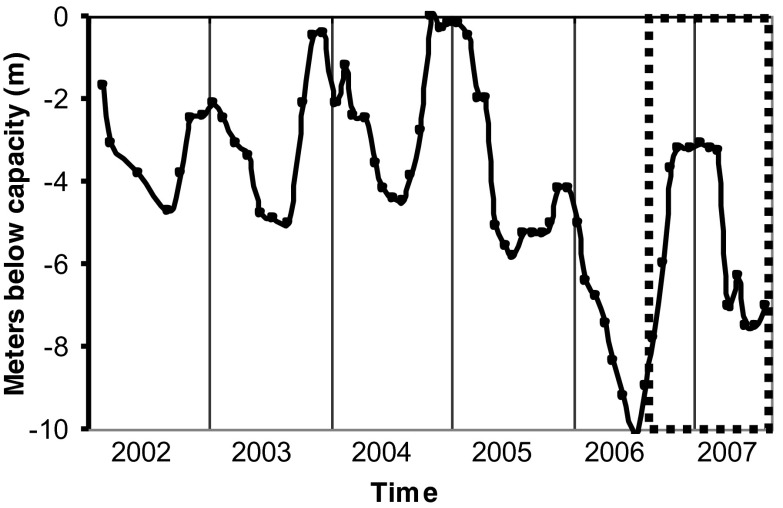
Water-level variations registered in the VB reservoir in 2002–2007. Level in meters below the capacity level. The *dotted box* marks the period of the community metabolism study

VB reservoir is in the central Mexican highlands (1,830 m. a.s.l, 19° 21′ 30′ N; 100°11′ 00″ W; Fig. 1), 127 km west of Mexico City Metropolitan Area (MCMA). VB is the largest reservoir of the Cutzamala System, which provides over one third of the water supply to MCMA (Ramírez-Zierold et al. 2010). At capacity (391 × 10^6^ m^3^), VB has a surface of 18.55 km^2^ and an average depth of 21.1 m (Merino-Ibarra et al. 2008). In average-to-rainy years (e.g., 2001–2004), the reservoir reaches capacity by the end of the rainy season (November). Seasonal variations in the balance between input from rivers (Fig. 1) and water withdrawal regularly cause a decrease of 4–5 m in the reservoir’s level during the summer (Fig. 2). However, during the drier years of 2006–2007 the depth of the reservoir decreased much more (by 10.1 m in 2006) and remained 3–5 m below capacity even during the winter.

Due to its depth, VB stratifies and behaves as a warm monomictic water body; it shows stable stratification from April through October and it is well mixed from November through February. A characteristic feature of VB is the persistence of a strong diurnal breeze, which makes it the most important sailing site in Mexico. The wind blows strongly (mean speed 7.4 m s^−1^, gusts up to 16.5 m s^−1^) from the dam (WNW; Fig. 1) to the valley tails from noon to sunset. Such strong winds drive oxygen concentrations rapidly to approximate saturation in the epilimnion, hindering the “free-water” monitoring of oxygen changes to assess metabolism (Merino-Ibarra et al. 2008). During the night and early morning, the wind is weaker (average 1.7 m/s) and blows in the opposite direction. Important thermocline displacements (up to 8 m) have been observed near the dam and tails of VB and attributed to stationary internal waves produced by this wind pattern (Monroy 2004). Merino-Ibarra et al. (2008) proposed that these waves might drive boundary mixing in VB, as has been reported for similar systems with internal waves (Ostrovsky et al. 1996; Zohary and Ostrovsky 2011). The occurrence of boundary-mixing events in VB is backed by (1) relatively high chlorophyll *a* concentrations (mean 4 μg L^−1^) in the hypolimnion (Merino-Ibarra et al. 2008) and (2) a steady increase in the hypolimnetic temperature throughout stratification (Ramírez-Zierold et al. 2010). Considering the regularity of this temperature increase, Merino-Ibarra et al. (2008) suggested that numerous small mixing events likely take place in VB during the stratification period, driving vertical flux of water and nutrients but without breaking the stratification.

Water quality in VB is mainly affected by eutrophication due to high nutrient loads derived from local sewage and wastewater from the watershed (116.8 t P year^−1^, and 557.1 t N year^−1^; Ramírez-Zierold et al. 2010). As a result, anoxia prevails in the hypolimnion during stratification (Monroy 2004; Ramírez-Zierold et al. 2010) and, although Mee’s (1988) “critical level” of eutrophication has not been reached yet, oxygen remains relatively low even during the circulation period (<65 % of saturation, Merino-Ibarra et al. 2008). The plankton composition of VB is also affected by eutrophication; cyanobacteria dominate during stratification and *Microcystis* blooms are frequent (Ramírez-García et al. 2002). During blooms observed in 2001, chlorophyll *a* reached up to 88 μg L^−1^ in the surface of VB (Merino-Ibarra et al. 2008). Zooplankton community is dominated by small-size species, particularly rotifers, as a consequence of cyanobacterial dominance (Nandini et al. 2008). In contrast, cladocerans, although not abundant overall, peak during the circulation period, when diatoms are dominant (Ramírez-García et al. 2002). Because of its steep margins and an anoxic hypolimnion, benthic life is scarce in VB (Merino-Ibarra et al. 2008).

## Methods

### Environmental variables

Temperature, conductivity, and oxygen were measured at 1 m intervals from surface to bottom at each station with a field probe (YSI 6600). Surface solar radiation (W m^−2^) during incubations was measured with a weather station (Vantage Pro, Davis Instruments). Secchi depth was measured with a standard disk. Water samples for nutrient analysis were filtered with 0.45 and 0.22 μm membranes and fixed with chloroform. A Skalar SanPlus segmented-flow analyzer was used with the standard methods adapted by Grasshoff et al. (1983) and the circuits suggested by Kirkwood (1994). Samples for chlorophyll *a* were filtered with 0.45 μm membranes (Millipore™), extracted with 90 % acetone and determined using a spectrophotometer and the Jeffrey and Humpfrey (1975) equations (for more details see Merino-Ibarra et al. 2008). Zooplankton data from Jiménez-Contreras (2009) were used to analyze biomass trends of the major zooplankton groups found in VB and explore their role in the regulation of primary production.

### Oxygen dynamics

Oxygen dynamics are a powerful tool to monitor metabolic changes in aquatic ecosystems (Staehr et al. 2012). Monitoring oxygen allows both detecting “critical eutrophication” (Mee 1988) and measuring community metabolism (Vollenweider et al. 1974). The utility of the diel oxygen technique for measuring lake metabolism has been recently outlined (Staehr et al. 2010). However, although “free-water” approaches can benefit from the use of automated sensors, atmospheric exchange can alter oxygen concentrations beyond possible correction in very windy systems (Merino-Ibarra et al. 2008). Monitoring oxygen evolution within enclosures avoids the issue of correcting for air-water exchange, and can therefore yield more reliable results in windy systems (Staehr et al. 2012). In particular, the in situ incubation of light and dark bottles yields reliable measures of the planktonic community production and respiration, particularly in highly productive systems (Vollenweider et al. 1974; Bender et al. 1987). Additionally, the low cost and conceptual simplicity of this method make it an easily available alternative for metabolism monitoring in productive systems throughout tropical countries, where equipment and resources are often limiting the environmental monitoring of aquatic ecosystems needed both for the better assessment of global rates and for local management.

However, the light and dark bottle method has its own limitations and implies several assumptions, which should be considered. Incubation experiments have two major problems (Bender et al. 1987): (1) processes that take place in bottles are not entirely equivalent to those that occur naturally, and (2) estimates of production and respiration rates from incubations often give ambiguous results and can fail to give complete community or ecosystem level rates. Bottle effects can include an overestimation of respiration rates due to bacterial growth; in fact, a variety of factors can increase *R* during daylight, including increased release of dissolved organic matter by primary producers in the light (with increased consumption by bacteria) and photorespiration (Howarth et al. 2013). Accordingly, the assumption of unaltered respiration rates in light and darkness applied to calculate *P*_g_ may be erroneous (Staehr et al. 2012). To deal with this, we assumed differential light and dark respiration rates (Geider and Osborne 1989), we incubated bottles in situ (see below) to minimize the effects of increased temperature and light and to keep conditions as real as possible.

Perhaps the problem of scaling is more critical: to obtain ecosystem level estimates of planktonic and benthic metabolic rates integrated over large spatial and time scales, many individual measurements must be summed and averaged, thus leading to potentially large propagation of errors and failure to capture spatial heterogeneity (Staehr et al. 2012). This issue can be partially dealt with by the calculation of the propagation of errors (e.g., Lehrter and Cebrian 2010, see below). To insure spatial representativeness, we incubated bottles at multiple depths and stations, so falling for another disadvantage of the method, which is that it is labor intensive.

Finally, bottles or chambers may not represent the entire ecosystem, which may introduce bias (Harmon et al. 2007). The exclusion of the grazing community and removal of the photosynthetic community from the turbulent processes of the water column can make the measured rates problematic (Staehr et al. 2012). It should be noted that this method does not capture benthic production and respiration, nor anaerobic respiration occurring in the aphotic zone, so it yields only an approximation to production and respiration of the ecosystem. In this case, metabolic measurements represent the activity of the water column community, and it should be so interpreted throughout this paper.

We incubated light and dark bottles in situ (sensu Vollenweider et al. 1974) at four sampling stations in VB (Fig. 1) to measure oxygen production and consumption every 4 weeks from July of 2006 to August of 2007. Three different sampling casts were done at each station to obtain three unaltered water samples from each of the levels sampled (0, 1, 2, 4, 6, and 8 m). From each sample, three oxygen bottles were filled (one for initial oxygen determination, one for light incubation and one for dark incubation). Special care was taken to totally avoid bubbling and contact with the atmosphere in all cases until sample fixation. The three light and three dark bottles from each level were incubated at their respective sampling depths during 5–7 h starting at noon. In the laboratory, dissolved oxygen concentrations were determined through triplicate titrations from each incubated bottle by the Winkler titration method (Grasshoff et al. 1983) to minimize and assess error.

Additionally, during the circulation, when oxygen was distributed throughout the water column, incubations were also conducted at the 12, 16, 20 and 28 m depths to measure the aerobic aphotic respiration rate and to allow assessment of the respiration over the full aphotic layer (*R*_aphotic_). The integration of *R*_aphotic_ to the respiration in the trophogenic layer (*R*) allowed calculating the total respiration of the reservoir (*R*_total_ = *R* + *R*_aphotic_) for the circulation period.

### Metabolism calculations

Gross primary production (*P*_g_), net production (*P*_n_), and community respiration (*R*) in the trophogenic layer were calculated using the oxygen change rate in the light and dark bottles, respectively, following Wetzel and Likens (1991) and Kemp and Testa (2011). Differences between initial and final oxygen concentrations were divided by the specific incubation time of each set of bottles. The base of the trophogenic layer was obtained linearly interpolating the PG rate to the depth where PG = 0 at each station and sampling date. To obtain area-based rates, scaled for the whole reservoir, volumetric rates were integrated over the trophogenic layer. To do this integration, the rates from each of the sampled levels were multiplied by the height of the water layer it represented (i.e., the volumetric rates from the 2 m level were multiplied by 1.5 m, from the 4 m level by 3 m, and so on). Because the area changes at the different depths of the reservoir, each layer was weighted proportionally to the fraction of the surface area that it encompassed, using the detailed hypsographic data obtained by Monroy (2004) for the VB reservoir. The same procedure was used when integrating *R*_aphotic_ rates to obtain *R*_total_.

To calculate diel rates, the hourly production rates were multiplied by the corresponding photoperiod for each sampling date at this latitude. In the case of diel respiration, the respiration during the night was also considered, multiplying the night-time hours by the dark respiration rate, which was calculated as 10 % of the *P*_g_ rate, following Geider and Osborne (1989). Productivity measurements are more frequently reported in terms of carbon than of oxygen. Therefore, to allow comparison with these data, we converted our oxygen rates to carbon, using the theoretical and most widely used conversion values (PQ = 1.3 and RQ = 1.0; Gazeau et al. 2005).

### Error propagation estimation

Metabolism calculations often involve relative small differences between large numbers, so it is critical to estimate the errors (and their propagation) associated with these means (Smith and Kemp 1995). Since our rates required only simple step-by-step calculations, we propagated the uncertainty throughout the calculations and the scaling using classical error propagation theory (Ku 1966). To obtain confidence intervals (CI) around our calculated variables, we used the standard error (SE) of the mean, which can be converted directly to CI by multiplying with the *t* value for the desired alpha and specific degrees of freedom (*df*) for each case (Lehrter and Cebrian 2010). Since our calculations only implied sums, products and quotients, we used the simplified equations proposed by these authors for simple step-by-step calculations, where SE_*W*_ is the SE of a function *W* calculated from the means of two or more variables (i.e., $W= f\left(\overline{U},\overline{V}\right)$):

1 $$ {\mathrm{SE}}_w=\sqrt{{\left( a{\mathrm{SE}}_{\overline{U}}\right)}^2+{\left( b{\mathrm{SE}}_{\overline{V}}\right)}^2} $$

Equation 1 applies for sums (i.e. $W= a\overline{U}\pm b\overline{V}$), and equation 2 for products or quotients (i.e. $W=\overline{U}\times \overline{V}\kern0.5em  or\kern0.5em \overline{U}/\overline{V}$).

2 $$ {\mathrm{SE}}_w=\sqrt{{\left(\frac{{\mathrm{SE}}_{\overline{U}}}{\overline{U}}\right)}^2+{\left(\frac{{\mathrm{SE}}_{\overline{V}}}{\overline{V}}\right)}^2} $$

These equations imply the assumptions that $\overline{U}$, $\overline{V}$ and their errors are uncorrelated and that their distributions are approximately normal, which are reasonable assumptions for our data. To obtain the propagated *df* for the calculated rater and there in their CI, we used the Welch-Satterwhaite formula (Ku 1966) as adapted and formulated by Lehrter and Cebrian (2010) for a more general form of $W= f\left(\overline{U},\overline{V}\right),\kern0.5em  W= f\left({\overline{W}}_1,{\overline{W}}_2,\dots, {\overline{W}}_j\right)$:

3 $$ d{f}_W=\frac{{\mathrm{SE}}_W^4}{{\displaystyle {\sum_{i=1}^j}_{d{f}_{{\overline{W}}_i}}^{c_i^4 S{E}_{{\overline{W}}_i}^4}}} $$

Equation 3 performs well for propagating *df* through calculations involving both directly measured and subjectively estimated variables, and also implies the assumptions that variables are approximately normal and ${\mathrm{SE}}_{{\overline{w}}_i}$ are statistically independent. Throughout our results, the calculated rates are reported including ± SE and *df* (in parenthesis) and CI are included in the plots.

## Results

### Spatial and temporal variation of *P*_g_, *R*, and *P*_n_

**Fig. 3:**
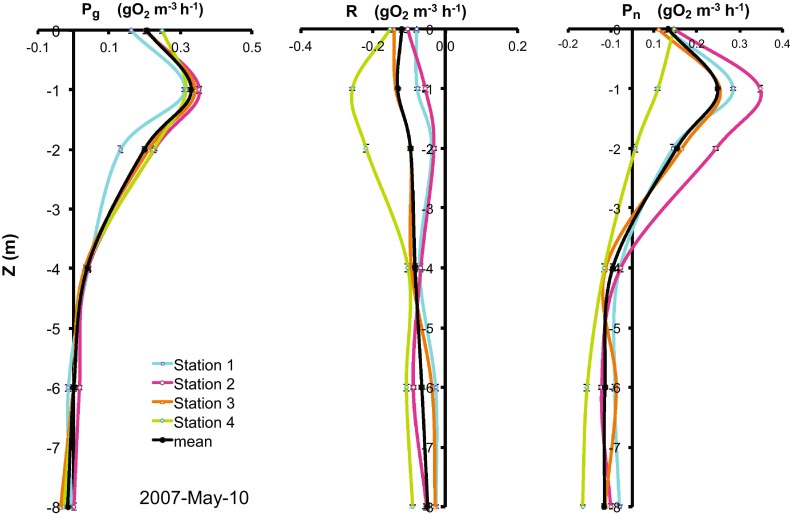
Vertical profiles of gross production (*P*_g_), respiration (*R*) and net ecosystem production (*P*_n_); mean and sampling stations 1–4 (gO_2_ m^−3^ h^−1^) in Valle de Bravo Reservoir during stratification (May 2007)

Oxygen incubation profiles showed coherent patterns in accordance with the expected vertical variation (i.e., *P*_g_ rates were always ≥0, while *R* rates were always negative and showed slight vertical variations, sustaining the reliability of the measurements (Fig. 3)). Maximum production occurred at 1 m (61 % of the cases) and at the surface (33 %). The base of the trophogenic layer (*P*_g_ > 0) was between 2 and 8 m, and its statistic mode was 6 m. These values are consistent with the Secchi depths measured in VB, which averaged 1.57 m (range: 0.95–2.75; Table 1). The vertically integrated rates of *P*_g_, *R*, and *P*_n_ did not show significant differences among the four sampled stations (one-way ANOVA; *p* = 0.49 for *P*_g_, *p* = 0.19 for *R* and *p* = 0.22 for *P*_n_; *n* = 52), which were therefore averaged to assess temporal variations of the reservoir as a whole.

**Table 1:** Gross production (*P*_g_), respiration (*R*), net production (*P*_n_) (means ± SE, with *df* in parentheses), *P*_n_:*P*_g_ ratio, soluble reactive silicon (SRSi), Secchi depth and chlorophyll *a* (Chl *a*) (means ± SE) in Valle de Bravo reservoir from August of 2006 to August of 2007

Period	*P*_g_	*R*	*P*_n_	*P*_n_:*P*_g_	Secchi (m)	Chl *a* (mg m^−2^)	SRSi (μmol L^−1^)
	(g O_2_ m^−2^ h^−1^)	(g O_2_ m^−2^ h^−1^)	(g O_2_ m^−2^ h^−1^)				
Annual mean	0.604 ± 0.097 (666.8)	−0.440 ± 0.069 (154.6)	0.169 ± 0.069 (166.6)	0.28	1.6 ± 0.01	150.6 ± 4.1	350 ± 8
Circulation^a^	0.491 ± 0.096 (616.5)	−0.533 ± 0.068 (154.2)	−0.033 ± 0.068 (154.1)	−0.07	2.1 ± 0.03	151.2 ± 11.2	538 ± 11
2006 Stratification^b^	0.617 ± 0.087 (410.4)	−0.161 ± 0.062 (102.5)	0.456 ± 0.062 (102.6)	0.74	1.2 ± 0.01	177.0 ± 23.5	295 ± 27
2007 Stratification^c^	0.764 ± 0.106 (817.2)	−0.509 ± 0.075 (204.3)	0.257 ± 0.075 (204.2)	0.34	1.5 ± 0.03	134.1 ± 7.5	157 ± 11

^a^October 2006 to March 2007

^b^August to September of 2006

^c^May to August of 2007

**Fig. 4:**
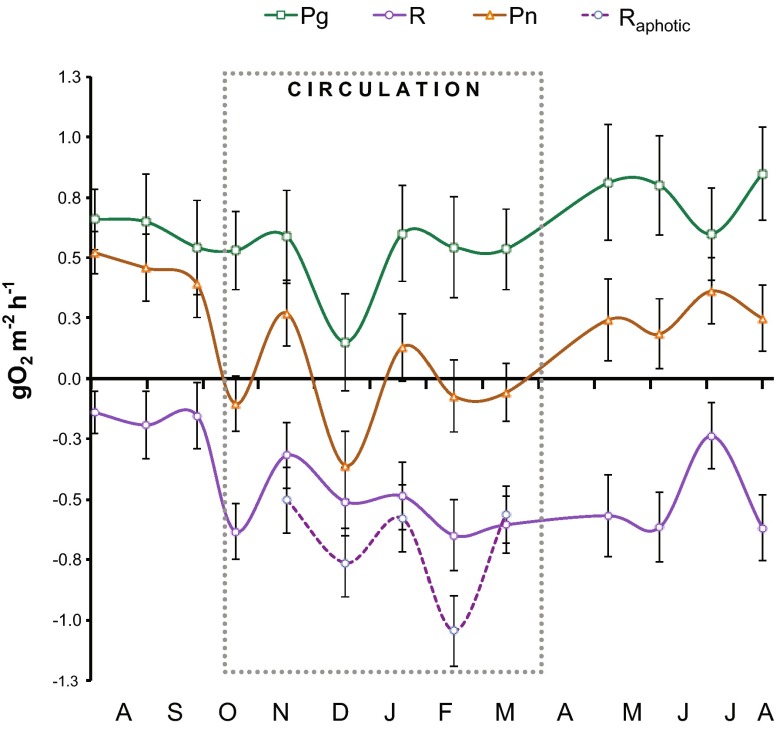
Time variations of gross primary production (*P*_g_), respiration (*R*) and net ecosystem production (*P*_n_) in the trophogenic layer of VB, and respiration in the aphotic layer (*R*_aphotic_) of VB reservoir from July 2006 to August 2007. *Error bars* depict the confidence intervals (CI) calculated from the propagated SE and *df* at *α* = 95 %. The *dotted box* encloses the circulation period

Metabolism measurements of the trophogenic layer of VB varied in time primarily in association with the stratification-circulation cycle of the reservoir (Fig. 4), so the metabolism data were summarized for stratification and circulation periods (Table 1). Oxygen consumption (*R*) increased from a mean of −0.16 g O_2_ m^−2^ h^−1^ during the 2006 stratification to −0.51 g O_2_ m^−2^ h^−1^ during circulation, and remained as high during the 2007 stratification. *P*_n_ averaged 0.46 gO_2_ m^−2^ h^−1^ in the 2006 stratification, dropped to slightly negative during circulation, and recovered to 0.26 g O_2_ m^−2^ h^−1^ in the 2007 stratification. In contrast, *P*_g_ showed less temporal variations around its 0.60 g O_2_ m^−2^ h^−1^ average. Only a sharp decrease in December 2006 stood out from the *P*_g_ temporal trend (Fig. 4) and pulled down the circulation average (0.49 g O_2_ m^−2^ h^−1^) below the stratification means. As the 95 % CI on Fig. 3 show, *P*_g_ for December was significantly different to the rest of the sampled months. Water clarity, as measured by Secchi depth, increased (from 1.2 to 2.1 m) during circulation (as observed previously, Merino-Ibarra et al. 2008), and decreased again in the 2007 stratification, but only to 1.5 m; consistent with this, chlorophyll *a* was 25 % lower during the 2007 stratification than during the 2006 one.

### Temperature, light, nutrients and zooplankton biomass

**Fig. 5:**
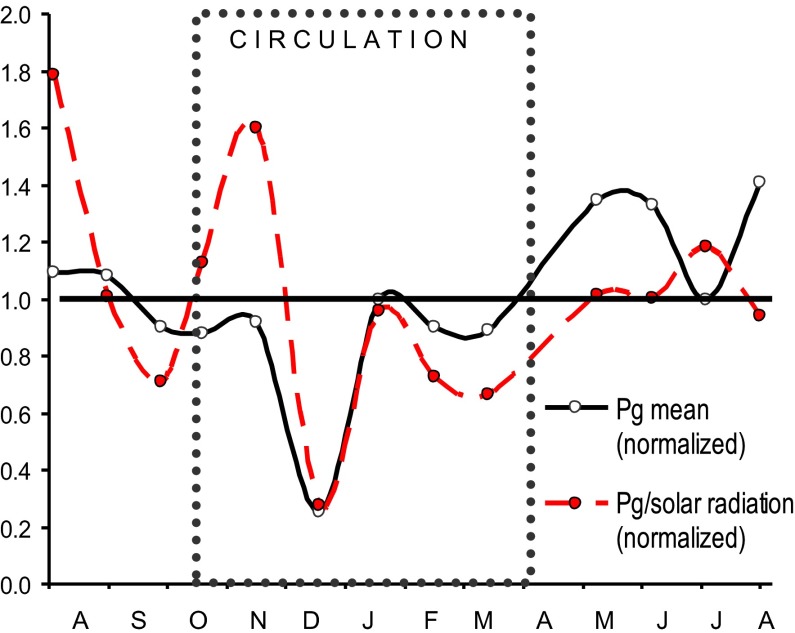
Gross production and *P*_g_/solar radiation (both normalized to their annual mean) in VB from July 2006 to August 2007. The *dotted box* marks the circulation period

*P*_g_ and *P*_n_ showed small but direct correlations with temperature (*r* ^2^ = 0.032 and *r* ^2^ = 0.057, respectively) similar to that found for marine phytoplankton (*r* ^2^ = 0.06) by López-Urrutia et al. (2006). However, in the case of *R*, temperature correlation was inverse (*r* ^2^ = 0.1029). The small (and insignificant, as shown above) variations of *P*_g_ among sampled stations decreased significantly when *P*_g_ rates were normalized by the solar radiation received during each incubation (Fig. 5), suggesting they derived from differences in light exposure, mainly due to cloud cover. Light-normalized *P*_g_ showed variations in the potential production throughout the year (Fig. 5), but the annual minimum (December) remained as such and hence it seems not attributable to light availability either.

**Fig. 6:**
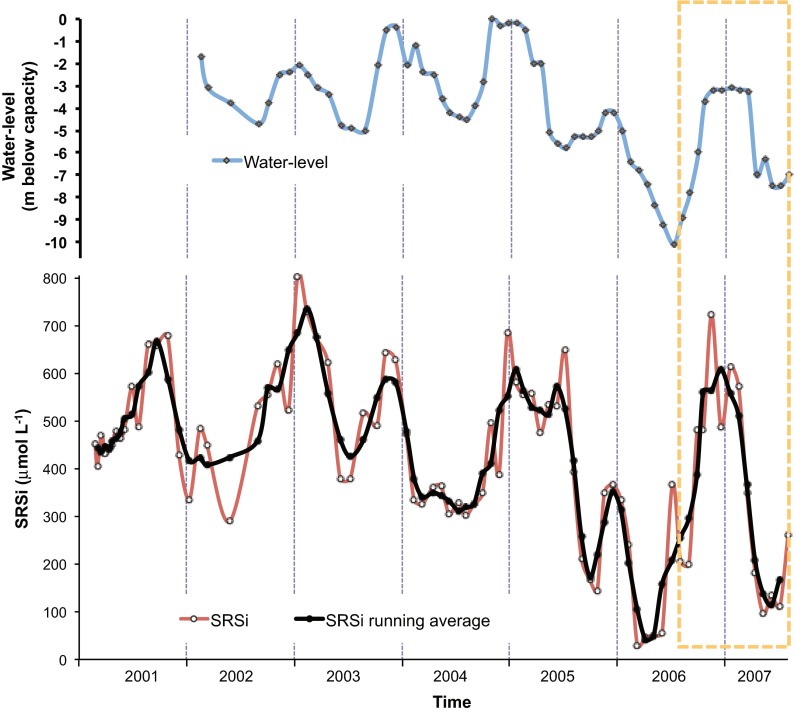
SRSi and its running average (5 data) from 2001 to 2007 in the trophogenic layer of VB (Merino-Ibarra, unpublished data). The rectangle marks the community metabolism study period

Nutrients within the production layer did not show clearly defined temporal trends, except for nitrate and silicate. Soluble reactive P (SRP) concentrations averaged 0.18 μmol L^−1^ and ranged from 0.08 μmol L^−^in January and February to 0.30 μmol L^−1^ in June and July. Ammonia averaged 3.4 μmol L^−1^ and was the most variable nutrient in time and among sampling stations, ranging 1.5–8.3 μmol L^−1^. In contrast, nitrate was very homogeneous among sampling stations (average 6.4 μmol L^−1^) and showed a marked time pattern: values peaked (>15 μmol L^−1^) during lake circulation (when nitrification of the ammonia that accumulated in the hypolimnion prevails) and were low (0.6–5.0 μmol L^−1^) during stratification. Dissolved inorganic N (DIN, average 10.4 μmol L^−1^) and followed a time pattern dominated by nitrate dynamics (maximum during circulation: 29 μmol L^−1^, and minimum in the stratification of 2007: 4.7 μmol L^−1^). Soluble reactive Si (SRSi, average 350 μmol L^−1^) was also homogeneous among stations but varied drastically in time: circulation concentrations were >350 μmol L^−1^, while lower values occurred during the stratifications, reaching a minimum of 96 μmol L^−1^ in May 2007 (Fig. 6).

**Fig. 7:**
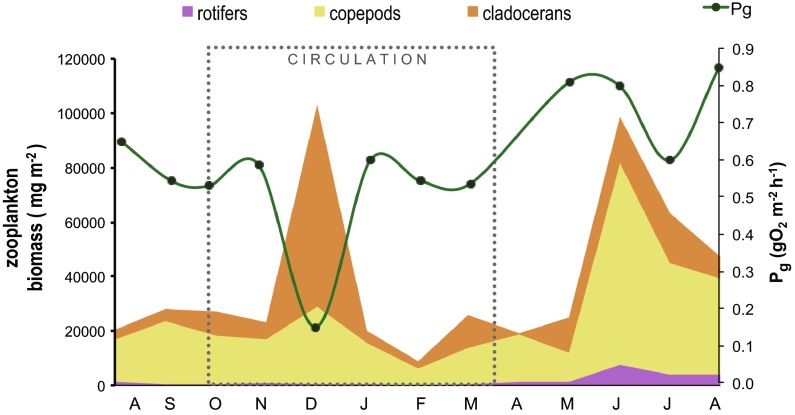
Time variations of zooplankton biomass (modified from Jiménez-Contreras 2009) and *P*_g_ in VB reservoir from July 2006 to August 2007. The *dotted box* marks the circulation period

Zooplankton biomass determined during the studied period is available for each of the major groups (Fig. 7): rotifers averaged 1,800 mg m^−2^, cladocerans 13,390 mg m^−2^, and copepods 24,100 mg m^−2^ (including nauplii stages). Rotifers and copepods (mainly Cyclopoidea) showed their minimum biomasses in February 2007 and their maximum in June and July 2007. In contrast, cladocerans showed their minimum in April 2007 and their maximum in December 2006, when a marked peak occurred. The biomasses proportion at this time (72 % cladocerans, 27 % copepods and rotifers only 1 %) deviated notably from that observed in the reference 4 years (2004–2007) database of Jiménez-Contreras (2009), when rotifers (8 % of the total zooplankton biomass) and copepods (59 %) were relatively more important, and cladocerans accounted only for 39 %.

### Metabolic balance

**Fig. 8:**
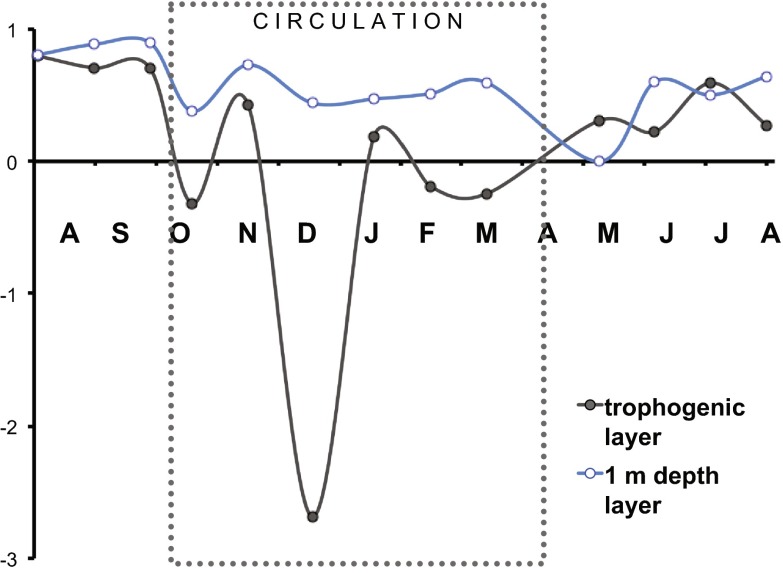
Net/gross production ratio (*P*_n_:*P*_g_) in the trophogenic layer (*solid circles*) and in the 1 m depth layer (*empty circles*) of VB from July 2006 to August 2007. The *dotted box* marks the circulation period

**Fig. 9:**
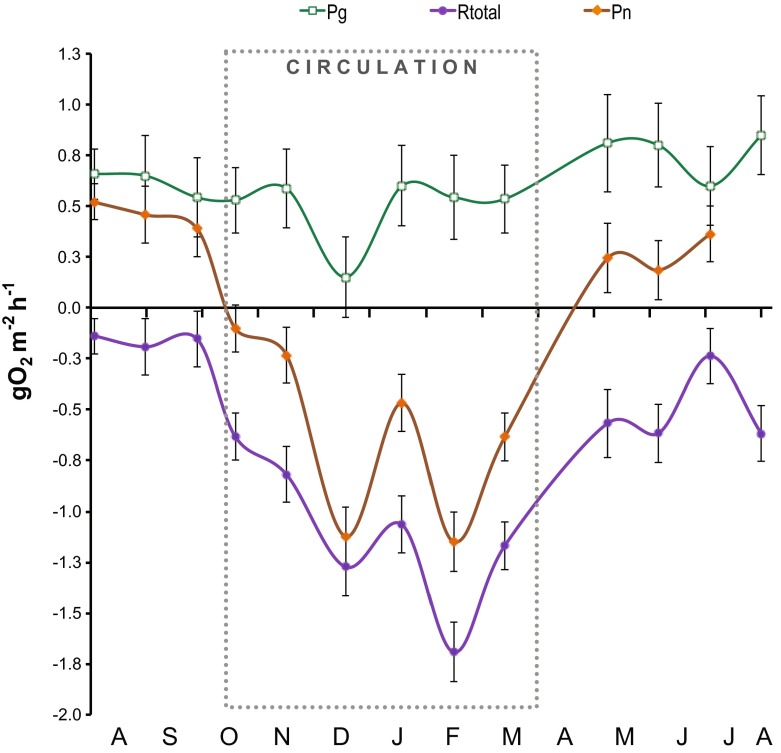
Gross production (*P*_g_), total respiration (*R*_total_ = trophogenic *R* + *R*_aphotic_) and recalculated net ecosystem production (*P*_n_) in the whole reservoir of VB from July 2006 to August 2007. Error bars depict the confidence intervals (CI) calculated from the propagated SE and *df* at *α* = 95 %. The *dotted box* marks the circulation period

Overall, the trophogenic layer of VB was autotrophic (*P*_n_ > 0, Table 1). However, respiration increases during the circulation period inverted the metabolic balance (Fig. 4). Respiration changes between the two stratifications sampled also reduced the potential biomass exportation (estimated by *P*_n_:*P*_g_, Fig. 8) of the trophogenic layer; *P*_n_:*P*_g_ decreased by half, from 0.74 in 2006 to 0.34 in 2007, mainly because *R* was much higher during the 2007 stratification. Vertical variation of the metabolic balance in VB was also shown by the comparison of *P*_n_:*P*_g_ ratios at the maximum production depth (1 m) with the whole trophogenic layer. At 1 m depth, *P*_n_:*P*_g_ was high and positive throughout the year (average: 0.62, range: 0.90 to 0.25), while *P*_n_:*P*_g_ for the whole trophogenic layer was smaller, and at times decreased sharply, particularly during the circulation period. This means that the surface layer (~1 m) of VB has a net autotrophic metabolism throughout the year, but important variations in respiration at the deeper part of the trophogenic layer can shift the metabolic balance of the full trophogenic layer toward net heterotrophy. Furthermore, when the respiration of the whole system (*R*_total_; Fig. 9) was assessed, through the addition of the *R*_aphotic_ measured during the circulation months (2.4 g C m^−2^ day^−1^) to the trophogenic respiration (*R*), the overall metabolic balance of VB turned out to be heterotrophic (*P*_g_:*R*_total_ = 0.70, Table 2).

**Table 2:** Gross production (*P*_g_), respiration (*R*) and *P*_g_:*R* ratios in lakes and reservoirs with advanced trophic state (ordered by mean *P*_g_). Means and *P*_g_:*R* were calculated from the original data when not reported and converted from O_2_ to C when necessary

Ecosystem	Reported trophic state	*P*_g_ (gC m^−2^ day^−1^)		*R* (gC m^−2^ day^−1^)		*P*_g_:*R* ^a^	Reference
		Average^b^	Range	Average	Range		
Ten reservoirs, South Brazil	Oligo to eutrophic	0.5	0.0008–0.92				Gianesella-Galvão 1984
Lake Biwa, Japan	Mesotrophic	0.8	0.21–1.48	0.7	0.35–1.07	1.19	Takahashi et al. 1995
Pond, Massachusetts		1.2	0.3–2.0	1.3	0.4–2.2	0.88	Cole and Fisher 1978
Lake Kinneret, Israel	Rich in nutrients	1.8	1.2–2.3				Berman et al. 1995
La Mariposa	Hypertrophic	1.8	0.9–2.6				González et al. 2003
Itchen River, England		2.4	0.1–4.6	4.1	1.4–6.7	0.58	Odum 1956
Pond, Texas	Eutrophic	2.6	0.4–4.7	2.1	0.7–3.4	1.49	Kelly et al. 1978
Valle de Bravo, Mexico	Eutrophic						
Stratification of 2006^c^		2.3		0.7		3.18	This study
Circulation^d^		1.7		2.2		0.74	This study
Stratification of 2007^e^		2.9		2.3		1.25	This study
Annual mean		2.2		2.1		1.02	This study
Annual mean (considering *R*_total_)		2.2		3.1		0.70	This study
Quebrada seca, Venezuela	Hypertrophic	2.7	1.8–3.5				González et al. 2003
Zeekoevlei, South Africa	Hypertrophic	2.8	1.2–4.3				Harding 1997
Oloiden, Kenya		3.1	1.58–4.54				Allanson 1990
Undrainable rural ponds, India	Hypertrophic	3.3	1.8–4.8	3.2	1.7–4.7	1.03	Olàh et al. 1986
Lake Conway, Florida		3.4	0.8–6	3.8	0.8–6.8	0.89	Fontaine and Ewel 1981
McIlwaine, Zimbabwe		3.8	1.64–6.03				Allanson 1990
Shahidullah Hall, Bangladesh		4.2	1.6–6.8	3.7		1.15	Khondker and Kabir 1995
Pao-Cachinche, Venezuela	Hypertrophic	4.7	2.5–6.8				González et al. 2003
Lake Victoria, Uganda		5.4	3.08–7.65				Mugidde 1993
Lake Xolotlán, Nicaragua	Hypertrophic	5.7	4.6–6.8				Erikson et al. 1998
German shallow lakes		5.9	0.9–10.9	5.3	0.6–10	1.11	Kalbe 1972
Silver springs, Florida		7.1	2.6–11.6	1.3	0.9–1.7	5.46	Odum 1956
Ten springs, Florida		9.9	0.2–19.6				Odum 1956

*R*_total_ = *R* + *R*_aphotic_ (measured during circulation)

^a^ *P*_g_:*R* were calculated from original data when not reported

^b^Means were calculated from original data when not reported

^c^August to September of 2006

^d^October 2006 to March 2007

^e^May to August of 2007

## Discussion

### Comparison of community metabolism of VB with other lakes and reservoirs

Although our measurements do not capture benthic production and respiration, they can be considered a good approximation to the ecosystem metabolism of VB because it is a deep reservoir with steep margins and an anoxic hypolimnion where benthic life is scarce (Merino-Ibarra et al. 2008). Overall, the trophogenic layer of VB showed high productivity and respiration during 2006–2007, when compared to other freshwater ecosystems. Its average gross production (2.2 g C m^−2^ day^−1^) ranks VB in intermediate among eutrophic and hypertrophic ecosystems from tropical and subtropical latitudes (Table 2). Respiration within the trophogenic layer of VB was also high (average 2.1 g C m^−2^ day^−1^), but not as much as *P*_g_ and therefore this layer of the reservoir was, on average, autotrophic (*P*_g_:*R* = 1.24). However, when the aphotic layer was included (by adding *R*_A_), the total *R* of the whole water column of the reservoir was much higher (average 3.1 g C m^−2^ day^−1^, similar to hypertrophic systems) showing that VB was in fact heterotrophic (*P*_g_:*R*_total_ = 0.70, *P*_n_ = −0.9 g C m^−2^ day^−1^, Table 2) during 2006–2007. This is consistent with the results of St. Louis et al. (2000), who find that reservoirs are exporting C to the atmosphere. Furthermore, the mean *P*_n_ obtained for VB as a whole is equivalent to a flux of 3.4 mg m^−2^ day^−1^ of CO_2_ from this reservoir to the atmosphere, which is quite close to the 3.5 mg m^−2^ day^−1^ mean flux of CO_2_ reported by St. Louis et al. (2000) for tropical reservoirs. This sustains that community metabolism monitoring using oxygen dynamics could be very useful to assess net C flux if respiration is measured throughout the entire oxygenated layer, as we did in VB. Our results also show that the propagated errors for the method we used were about one order of magnitude smaller than the calculated rates (SE, Table 1) and the 95 % confidence limits (Figs. 3 and 8) allowed verifying the existence of significant differences among most of the rates measured.

### Environmental variables and zooplankton

Simultaneously monitoring of environmental variables and zooplankton biomasses helped assess their effects on the metabolic balance in VB. Our data did not show evidence of nutrient limitation (N, P, or Si) on primary production in VB, as expected in a eutrophic system. Although the N:P ratio (which averaged 88 and remained >16) indicated the possibility of P limitation, SRP was generally above the P limitation threshold (0.1 μmol L^−1^) proposed by Reynolds (1999). The only exception occurred in January (0.08 μmol L^−1^), when production limitation seems unlikely since *P*_g_ was high and above the annual average. Si limitation is also unlikely in VB, since SRSi concentrations were high and always above the limitation threshold, as in previous years (Merino-Ibarra et al. 2008).

The main variation of primary production we observed in VB, the sharp decrease of December 2006 (Fig. 4), was not apparently caused by radiation changes either (Fig. 5). We attribute this sharp *P*_g_ decrease to grazing. The observation of a concurrent five-fold zooplankton biomass peak sustains this possibility (Fig. 7). This peak was mainly due to cladocerans, which rose 1,200 % when *P*_g_ fell to its minimum. While cladocerans regularly peak during mixing in VB (Ramírez-García et al. 2002), the peak of December 2006 outstands as the highest attained by this group in the 4-year period (2004 to 2007) during which zooplankton has been studied in VB (Jiménez-Contreras 2009; Jiménez-Contreras et al. 2009; Nandini et al. 2008).

We propose that these changes in VB were favored by the extreme WLFs that started in the second half of 2006. A similarly drastic zooplankton biomass peak occurred again as the water level reached its minimum (almost −8 m from capacity) during the high WLF of 2007. Although this peak occurred during stratification, all the zooplankton groups, and particularly copepods, increased their biomass considerably during the 2007 WLF (Fig. 7). Jiménez-Contreras (2009) also outlined the anomalous zooplankton biomass increases observed during the high WLF years of 2006 and 2007 in comparison with the previous years (2004 and 2005), when WLF were small and water level remained near the reservoir’s capacity (Fig. 2).

### Community metabolism and WLF

WLF increased substantially in VB during 2006 and 2007, when the summer minimum levels were 3–5 m lower than in previous years. Although water level decreased more during the stratification period of 2006 than in 2007, our sampling started during the late stratification, when the level was rising rapidly again (Fig. 2), so our data probably do not reflect the effect of the intense WLF during this stratification. In contrast, our sampling period covered well the peak of the WLF during the stratification of 2007, so the metabolism measurements of the stratification of 2007 are expected to represent better the effect of extreme water-level reduction during stratification in VB.

One of the main ways WLF can affect water bodies is by changing their hydrodynamic processes, particularly in deep stratified systems (Zohary and Ostrovsky 2011). In turn, water movements, and mixing in particular, are among the main environmental variables affecting plankton dynamics (Naselli-Flores 1998; Reynolds 2006). This is sustained in VB by the fact that the annual mixing regime was the main factor affecting its community metabolism and structure, as found for other deep well-stratified reservoirs (Becker et al. 2009). During the circulation period, *R* increased drastically (by three-fold, Table 2) and the trophogenic layer of the reservoir became net heterotrophic (*P*_n_ < 0, Table 1). Continuous mixing at this time likely drives the increase in *R* by allowing the oxidation of organic matter and ammonia accumulated in the hypolimnion. We attribute the physiologically unexpected inverse correlation of *R* with temperature to this mixing-driven intensification of *R* that occurs during the circulation (and also colder) months. We pose therefore that this correlation is due to what is happening in the ecosystem as a whole, rather than on the organisms physiological scale, where positive correlations are expected (e.g., López-Urrutia et al. 2006). The absence of a positive correlation has also been found by others (Howarth et al. 2013), who also attributed this to the scale of comparison.

Following from the above, a decrease in *R* should follow the establishment of a new stratification (which regularly occurs around March in VB, Ramírez-Zierold et al. 2010), as reduced substances are again isolated in an anoxic hypolimnion. However, during the early stratification of 2007, *R* did not diminish and remained as high as during circulation (Tables 1 and 2). We propose that this unexpected pattern could derive from a relative intensification of boundary-mixing events during the first half of 2007. This was predicted to occur in VB when the water level decreases during stratification, as internal waves oscillate closer to the bottom of the reservoir (Monroy 2004; Merino-Ibarra et al. 2008), and has been observed in other stratified systems (Ostrovsky et al. 1996; Zohary and Ostrovsky 2011).

There are other evidences of enhanced mixing in VB during the 2007 stratification: (1) after complete oxygen depletion had been reached in April, the oxygen profiles of the May–August samplings (not shown), again exhibited substantial hypolimnetic oxygen concentrations (average of 0.4 mg O_2_ L^−1^). In the absence of a replenishment mechanism (mixing), total consumption of this oxygen would only take 4.7–13.2 h at the *R* rates measured in these months. Since we found oxygen in consecutive monthly samplings in May–August, its replenishment must have occurred with a near-daily frequency. This pattern supports the occurrence of small magnitude (not enough to destroy stratification) mixing events with high, quasi-diurnal frequency, as would be expected if they were caused by diurnal internal waves.

(2) SRSi is regularly high in VB due to the volcanic nature of the VB basin (~500 μmol L^−1^, Merino-Ibarra et al. 2008). However, after 2005, as high WLF began SRSi decreased sharply in the trophogenic layer of VB, particularly during the stratification periods. In 2007, SRSi diminished from 614 μmol L^−1^ in January to only 96 μmol L^−1^ in May and remained similarly low the following months (Fig. 6). Sharp SRSi decreases are frequently due to uptake by intense diatom development (e.g., Brown et al. 2003; Reynolds 2006). A ten-fold diatom biomass increase occurred in VB from January to May (Jiménez-Arreola 2010). Because mixing favors diatoms over other algal groups (Hecky and Kling 1981; Huisman and Weissing 1994; Huisman et al. 2004), the observed SRSi decreases and intensive diatom growth indicate the intensification of mixing events in VB during the stratification and high WLF of 2007.

All together, these observations sustain the significant hydrodynamic and ecological effects that WLF can cause in VB. In particular, the persistence of high R during the early 2007 stratification shows how a sharp level decrease can affect the community metabolism and shift it from autotrophy to heterotrophy. This result is coherent with the conclusion of Zohary and Ostrovsky (2011), that one of the important effects of extreme low water levels is the promotion of internal nutrient cycling. Although we did not measure here the change in the nutrient internal load directly, the increase in *R* we found implies an intensification of nutrient recycling. This result is also important for the reassessment of the contribution of reservoirs and lakes to the global carbon cycle (St. Louis et al. 2000; Tranvik et al. 2009), as it shows that level abatement could cause a decrease in the carbon sequestration by deep stratified systems.

The dramatic changes in zooplankton composition and the increase of diatoms during lower level periods in VB sustain the findings of Naselli-Flores and Barone (1997), Mac Donagh et al. (2009), Wang et al. (2011) and Zohary and Ostrovsky (2011), that WLF can also drive important community structure changes. Recent detailed phytoplankton studies in VB under intense WLF (Valeriano-Riveros et al. 2014), confirm that composition sharply shifts, showing a significant decrease in noxious algae and an increase of planktonic diatoms as water level decreases, thus ameliorating the water quality of the reservoir and reducing the threat of toxic cyanobacteria blooms.

The application side to this is that, as proposed by Geraldes and Boavida (2005), properly regulated WLF could be a useful management tool for VB and other eutrophic systems, where cyanobacteria dominance is one of the main concerns.

Specific management strategies could be to either: (1) keep the water level of the reservoir low during the stratification, when blooms of noxious algae (mainly cyanobacteria) are favored by the lack of mixing, or (2) decrease water level sharply at times when blooms are expected or already occurring. In a water supply system, the main condition for these actions would be that they could be coupled with usage patterns and water availability, which in turn will change as a function of climate.

The monsoonal climate regime of VB, with pronounced dry (October–May) and rainy (June–September) seasons (Valeriano-Riveros et al. 2014) favors this management strategies, at least during the first half of the stratification, since water input is low just at the right time for helping decrease water level for the stratification period. The interconnection of seven reservoirs in the Cutzamala water supply system would also help using water level as a management tool in VB, since managers can decide from which reservoir water is withdrawn on a daily basis, and pumping back is also possible (Ramírez-Zierold et al. 2010). Since the rest of the reservoirs are not used as intensively as VB for tourism and recreation, subordinating their water level to the management needs for VB would be feasible.

Climate change predictions for central Mexico are still variable, particularly for rainfall changes (Karmalkar et al. 2011), but in terms of global climate change scenarios, WLF are expected to magnify and extend due to the increased occurrence of extreme events (flooding, extended droughts) and increased anthropogenic use (Zohary and Ostrovsky 2011). Increasing variability in water availability will also likely mean a stronger competition between different human demands associated to water bodies, therefore increasing the importance of understanding the role of WLF on aquatic ecology and water quality.

Although the effects and potential application of WLF for management purposes will likely vary among different climatic regions and water bodies, our findings may help identify the possibilities of this alternative elsewhere. In developing countries, many water sources lack of adequate monitoring and efficient management strategies. These systems offer an opportunity both to further monitor and understand the effects of WLF and also to explore the possible applications for their management.

## Summary and conclusions

Our results confirm the usefulness of monitoring oxygen dynamics using enclosures to assess community metabolism in windy systems. Following the evolution of *P*_g_, *R*, and *P*_n_ during high WLF in the deep reservoir of VB helped identify the effect of extreme low water levels on the planktonic community metabolism and structure. The system shifted from net autotrophy to net heterotrophy during circulation and furthermore when the aphotic respiration was considered, resulting in a high total respiration. Low water levels are posed to affect the system mainly through mixing enhancement, even during the stratification, when boundary-mixing events are driven by the wind in VB. As proposed for deep systems (Zohary and Ostrovsky 2011), WLF likely enhance nutrient recycling in VB. Changes observed in the planktonic community in VB include zooplankton biomass increases of the bigger-size groups, cladocerans and copepods, and also diatom proliferation that matched with strong silicon decreases and cyanobacteria decreases during low water-level periods. These results may be applied to water management, using water level to reduce the threat of noxious blooms. Multiple interconnected reservoir systems, like in VB, and monsoonal climatic regimes may favor the application of this option elsewhere.
